# Evaluation of Four Genetic Variants in Han Chinese Subjects with High Myopia

**DOI:** 10.1155/2015/729463

**Published:** 2015-01-05

**Authors:** Zimeng Ye, Huaichao Luo, Bo Gong, Ying Lin, Ping Shuai, Pu Wang, Changning Ye, Zhenglin Yang, Wanjun Wang, Yi Shi

**Affiliations:** ^1^College of Life Science and Engineering, Southwest Jiaotong University, 111, First Section, Northern Second Ring Road, Chengdu, Sichuan 610031, China; ^2^Sichuan Provincial Key Laboratory for Human Disease Gene Study, Hospital of the University of Electronic Science and Technology of China and Sichuan Provincial People's Hospital, 32 the First Ring Road West 2, Chengdu, Sichuan 610072, China; ^3^Department of Laboratory Medicine, Sichuan Provincial People's Hospital, 32 the First Ring Road West 2, Chengdu, Sichuan 610072, China; ^4^School of Medicine, University of Electronic Science and Technology of China, 32 the First Ring Road West 2, Chengdu, Sichuan 610072, China

## Abstract

High myopia is one of the leading causes of blindness worldwide. However, the exact etiology of high myopia remains unraveled despite numerous attempts of elucidation. Previous genome-wide association study (GWAS) has revealed that four single nucleotide polymorphisms (SNPs), including rs2969180, rs1652333, rs9307551, and rs7837791, were associated with high myopia in Caucasians. The present study was conducted to investigate whether these genetic variants were associated with high myopia in Han Chinese. These four SNPs were genotyped by SNaPshot method in a Han Chinese cohort composed of 827 patients with high myopia and 988 healthy controls. Among the SNPs genotyped, only rs9307551 was found to be significantly associated with high myopia in this study. Carriers of rs9307551A allele, AA, and AC genotypes had an increased risk of high myopia (OR = 1.33, 95% CI 1.14–1.54; OR = 1.75, 95% CI 1.28–2.38; OR = 1.59, 95% CI 1.24–2.01, resp.). Interestingly, when split by gender, the association between rs9307551 and high myopia proved to be gender-specific with significance observed only in females but not males. These findings suggested that the SNP of rs9307551 showed a gender-specific association with high myopia in the Han Chinese population. In addition, *LOC100506035*, a lincRNA gene, might play a crucial role in the susceptibility to high myopia.

## 1. Introduction

Myopia is a common cause of visual impairment worldwide [1], with a prevalence of 33.1% in the USA [2], 16.4% in Australia, and 26.6% in the Western Europe [3]. The prevalence of myopia in Asian populations is up to 40–70% [4–6]. In mainland China, almost 43.9% of the population suffer from myopia [7]. Low myopia (with a refractive error ⩾ −3.0 D) and medium myopia (with a refractive error between −6.0 and −3.0 D) are primarily physiological, whereas high myopia (with a refractive error ⩽ −6.0 D) is one of the leading causes of blindness in the world. High myopia (HM) is associated with pathologic ocular changes, such as myopic macular degeneration, glaucoma, and retinal detachment [8–10]. In China, the prevalence of HM is 4.1% [7] and is much higher in high school and university students [11].

Previous studies have indicated the involvement of genetic and environmental factors in the pathogenesis of myopia; genetic factors play a major role in the development of high myopia [12, 13]. Recently, the application of genome-wide association study (GWAS) has revealed several novel single nucleotide polymorphisms (SNPs) susceptible to HM in the Caucasian populations, including rs2969180, rs1652333, rs9307551, and rs7837791 [14]. Here, in order to investigate whether these SNPs were associated with high myopia in Han Chinese population, we conducted a case-control study in a Han Chinese cohort composed of 827 unrelated individuals with high myopia and 988 unrelated normal controls.

## 2. Materials and Methods

### 2.1. Study Population

This study was approved by the Institutional Review Board of Sichuan Provincial People's Hospital. All participants gave their written informed consent to participate in the study. The study was performed under the principles of the Declaration of Helsinki. Patients with HM and normal matched controls were recruited at the Ophthalmology Clinic of Sichuan Provincial People's Hospital. All participants went through a standard examination protocol. In this study, the diagnosis for high myopia required the spherical equivalent less than or equal to −6.0 DS in both eyes and the axial length of the eye globe greater than or equal to 26.0 mm. Individuals were excluded from the study if they had undergone ocular procedures that might alter refraction or if they had other symptoms besides HM. For the controls, the criteria were no evidence of disease in either eye and a spherical equivalent from −1.0 to +1.0 DS. The controls were from the same district as the cases. All of the participants in this study were Han Chinese. In total, 827 HM patients and 988 normal controls were recruited. Demographic and clinical information of the patients and controls were listed in Table 1.

### 2.2. Selection of SNPs

In 2013, the Consortium of Refractive Error and Myopia (CREAM) performed a genome-wide meta-analysis of multiancestry cohorts and identified 16 new loci for refractive error in individuals of European ancestry, of which 8 were shared with Asians. Combined analysis identified 8 additional associated loci, of which 5 were shared with Asians [14]. In this study, we chose 4 of these 13 shared SNPs and planned to investigate the other 9 in future studies. The 4 SNPs we selected were rs2969180 (intronic* SHISA6 *gene), rs1652333 (24 kb 5′ of* CD55* gene), rs9307551 (33 kb 5′ of* LOC100506035 *gene), and rs7837791 (147 kb 3′ of* TOX *gene).

### 2.3. Genotyping

Venous blood of each subject was drawn and collected in an EDTA-containing tube. Genomic DNA was extracted from the blood using a Gentra Puregene Blood DNA kit (Gentra, Minneapolis, MN). SNP genotyping was conducted by the dye terminator-based SNaPshot method, with success rate and accuracy greater than 99%, as judged by random regenotyping of 10% of the samples in the subject group.

### 2.4. Statistical Analysis

The Hardy-Weinberg equilibrium (HWE) for each SNP was tested with a standard observed-expected chi-square test (*χ* ^2^ test). The overall association study was adjusted for age and gender with binary logistic regression, whereas gender-stratified analysis was adjusted for age. The genetic association analysis was carried out by constructing 2 × 3 tables of the genotype counts and 2 × 2 tables of the allele counts for each SNP in both patient and control groups. Subsequently, Pearson *χ* ^2^ statistics were calculated and *P* values were computed by comparing the statistic to a *χ* ^2^ distribution with 1 or 2 degrees of freedom for the allelic and genotypic tests. Odds ratio (OR) corresponding to 95% CI was used to assess the strength of association between the SNPs and susceptibility to high myopia. The significance of the pooled OR was determined by the *Z*-test. All statistical analyses were conducted using SPSS (version 17.0) software, and all results were considered to be statistically significant when *P* < 0.05.

## 3. Results

All of the four SNPs were successfully genotyped, and the genotype distributions were within Hardy-Weinberg equilibrium in both patient and control groups (*P* > 0.05, Table 2). Among the SNPs genotyped, rs9307551 was found to be significantly associated with high myopia. The minor allele (A) frequency (MAF) of rs9307551 was 0.445 in controls and 0.505 in patients, and the adjusted allelic *P* value was 2.46 × 10^−4^, with an adjusted odds ratio of 1.33 (95% confidence interval, 1.14–1.54). The *P* value of rs9307551 still showed significant association with HM after the Bonferroni correction (corrected *P* = 9.84 × 10^−4^). No significant association was observed between HM and the other three SNPs (*P* > 0.05, Table 2).

We further investigated the association between HM and rs9307551 by using different genetic models. The genotype distribution of rs9307551 is shown in Table 3. The frequency of CC genotype was significantly higher in the controls than that in HM patients (31.2% in controls and 23.6% in patients). The results suggested that subjects carrying rs9307551AC, rs9307551AA, and rs9307551AA + AC were more likely to suffer from HM than those carrying rs9307551CC (additive 1 model: *P* = 0.012, adjusted OR [95% CI] = 1.49 [1.15–1.94]; additive 2 model: *P* = 3.37 × 10^−4^, adjusted OR [95% CI] = 1.75 [1.28–2.38]; dominant model: *P* = 2.12 × 10^−4^, adjusted OR [95% CI] = 1.59 [1.24–2.01]). In addition, the recessive model indicated that subjects bearing rs9307551AA had a 1.34-fold (95% CI: 1.04–1.73) increased likelihood of HM compared to those who harbor rs9307551AC and rs9307551CC. In the meantime, different genetic models were also applied to analyze the association between rs2969180, rs1652333, and rs7837791 and HM. Still, no significant association was found (data not shown).

In order to study whether rs9307551 showed significant association only with one gender, we split the subjects by gender and investigated the association by using different genetic models (Table 4). Interestingly, our data indicated that the association between rs9307551 and high myopia was gender-specific. The association was only observed in females but not males. We also found that the association was not only maintained but also strengthened in the female group, despite the reduced sample size. The allelic model showed that rs9307551A was the risk allele of HM (OR [95% CI] = 1.47 [1.19–1.84], *P* = 2.86 × 10^−4^). Females with rs9307551AC, rs9307551AA, and rs9307551AA + AC, respectively, had a 1.66-fold (95% CI: 1.28–2.45, *P* = 0.012), 2.12-fold (95% CI: 1.39–3.24, *P* = 4.65 × 10^−4^), and 1.95-fold (95% CI: 1.40–2.72, *P* = 7.99 × 10^−5^) increased likelihood of HM compared to those females carrying rs9307551CC. Furthermore, the recessive model showed also that female subjects bearing rs9307551AA had a 1.41-fold (95% CI: 1.00–2.01) increased likelihood of HM compared to those females with rs9307551AC and rs9307551CC. None of these effects, however, was observed in males.

## 4. Discussion

In 2013, the Consortium of Refractive Error and Myopia (CREAM) presented the results from the largest international genome-wide meta-analysis and reported 24 new loci associated with myopia in the Caucasian populations, including rs2969180, rs1652333, rs9307551, and rs7837791 [14]. As shown in the results of the CREAM study [14], the “A” allele of rs9307551 showed decreasing effect with spherical equivalent. In this study, our results demonstrated that the “A” allele of rs9307551 had an increased risk of high myopia (Tables 2 and 3). And taking the results of these two studies together, we concluded that the direction of the effect was the same in CREAM as in our study. However, no significant association was found in the other three SNPs in this study. The sample size in this study was much smaller than that in CREAM study. This might be one of the reasons why we cannot replicate the results of CREAM. And other reasons could be allele frequency differences, different LD patterns between populations, and ethnic differences.

On the other hand, gender-specific genetic effects have been observed in other traits [15, 16]. In this study, our results suggested that the genetic variant located at 33 kb 5′ of* LOC100506035 *gene was associated with HM in a gender-specific way. This association was only found in females but not in males. However, there have been too few studies addressing the effect of gender on the risk of HM. In 2002, Saw et al. pointed out that myopia was associated with being male in Singaporean Chinese children [17]. But, in 2012, You et al. noted that myopia in school children in Greater Beijing was associated with female gender [18]. As a result, the gender-specific association seen in our study could provide insights into the understanding of the etiology of HM in the future. Gender-specific associations might result from gene-gene or gene-environment interactions. It is possible that women are exposed to an environment which interacts with the gene. These results underlie the potential importance of analyzing HM data both with and without gender as a stratifying factor.

*LOC100506035 *gene has also been called long intergenic nonprotein coding RNA 989* (LINC00989). *Large intergenic noncoding RNAs (lincRNAs) have recently been identified as a kind of new RNA molecule in gene regulation [19, 20]. The lincRNAs, which proved functional, showed diverse biological activities, such as involvement in X chromosome inactivation and regulation of gene expression in cancer cells, stem cells, and development [21–24]. More than 30% of lincRNAs were estimated to be associated with chromatin-modifying complexes, such as co-REST and PRC2, and subsequently target those complexes to specific genomic regions [19]. HOTAIR, a lincRNA which is part of the* Hox* gene cluster, proved to be related to breast cancer progression [25]. And ANRIL, a lincRNA regulating CDKN2A and CDKN2B, was reported to be associated with atherosclerosis [26]. However, despite the potentially broad functional impact of lincRNAs in regulation of gene expression, no lincRNAs have been reported to be related to HM. In this study, the data showed that a genetic variant of a lincRNA gene may, at least in part, play a crucial role in the development of HM in Han Chinese population. This may facilitate the study of lincRNAs and provide a new direction to investigate the genetic mechanism implicated in HM.

In summary, our data revealed the gender-specific association between rs9307551 and HM in Han Chinese population. Female carriers of rs9307551 risk allele (A) were more likely to suffer from HM. Our results suggested the potential importance of analyzing HM data both with and without gender as a stratifying factor. In addition, our results suggested that a genetic variant in a lincRNA gene (*LOC100506035*) increased susceptibility to high myopia in Han Chinese.

## Figures and Tables

**Table 1:** Demographic and clinical characteristics of HM patients and controls in this study.

Group	Number	Average age (years)	Gender		Refractive errors (diopter)		Axial length (mm)	
			Male	Female	OD	OS	OD	OS
Patient	827	35.30 ± 16.55	318	509	−11.34 ± 4.65	−11.04 ± 4.49	28.16 ± 2.24	27.97 ± 2.41
Control	988	49.68 ± 17.22	581	407				

±: standard deviation; OD: right eye; OS: left eye.

**Table 2:** Association between HM and four SNPs (rs2969180, rs1652333, rs9307551, and rs7837791) in a Han Chinese population.

SNP	Chromosome	Position	Gene	Minor allele	MAF		*P*_HWE		Allelic *P* ^*^	Corrected *P* ^&^	OR (95% CI)^**^
					Patient	Control	Patient	Control			
rs2969180	17	11407900	*SHISA6 *	G	0.472	0.483	0.208	0.722	0.581	1	1.04 (0.90–1.22)
rs1652333	1	203858855	*CD55 *	T	0.452	0.420	0.615	0.707	0.936	1	1.00 (0.85–1.16)
rs9307551	4	80530670	*LOC100506035 *	A	0.505	0.445	0.302	0.580	2.46 × 10^−4^	9.84 × 10^−4^	1.33 (1.14–1.54)
rs7837791	8	60179085	*TOX *	T	0.478	0.473	0.888	0.064	0.605	1	1.04 (0.90–1.21)

^*^Allelic *P* value has been adjusted by age and sex.

^**^ORs (95% CI) have been adjusted by age and sex and were determined by the *χ* ^2^ test, patients versus controls.

^&^Corrected *P* = allelic *P*  ×  4 (the number of genotyped SNPs).

SNP: single nucleotide polymorphism; MAF: minor allele frequency; HWE: Hardy-Weinberg equilibrium; OR: odds ratio.

**Table 3:** The SNP of rs9307551 in the *LOC100506035* gene was associated with HM in this study.

Group	Genotype (*n*%)			*P*_HWE	Model	Crude OR (95% CI)	Adjusted OR (95% CI)	Adjusted *P*
	AA	A/C	CC					
Control	200 (20.3)	479 (48.5)	308 (31.2)	0.580		1	1	
Patient	206 (25.7)	408 (50.7)	190 (23.6)	0.302	Allelic	1.24 (1.10–1.41)	1.33 (1.14–1.54)	2.46 × 10^−4^
					Additive 1	1.40 (1.13–1.73)	1.49 (1.15–1.94)	0.012
					Additive 2	1.54 (1.20–1.98)	1.75 (1.28–2.38)	3.37 × 10^−4^
					Dominant	1.44 (1.18–1.76)	1.59 (1.24–2.01)	2.12 × 10^−4^
					Recessive	1.24 (1.00–1.52)	1.34 (1.04–1.73)	0.025

Crude ORs (95% CI) were determined by the *χ* ^2^ test, patients versus controls. Adjusted ORs (95% CI) and adjusted *P* values were obtained by adjusting for age and sex.

Genotype (AA/AC/CC) analyses were conducted for the allelic model (A compared with C), additive model 1 (AC compared with CC), additive model 2 (AA compared with CC), dominant model (AA + AC compared with CC), and the recessive model (AA compared with AC + CC).

**Table 4:** The association between HM and rs9307551 was gender-specific.

Gender	Group	Genotype (*n*%)			*P*_HWE	Model	Crude OR (95% CI)	Adjusted OR^#^ (95% CI)	Adjusted *P* ^#^
		AA	A/C	CC					
Female	Control	82 (20.3)	191 (47.3)	131 (32.4)	0.417		1	1	
	Patient	133 (26.9)	259 (52.4)	102 (20.7)	0.241	Allelic	1.45 (1.20–1.74)	1.47 (1.19–1.84)	2.86 × 10^−4^
						Additive 1	1.74 (1.27–2.40)	1.66 (1.28–2.45)	0.012
						Additive 2	2.08 (1.43–3.04)	2.12 (1.39–3.24)	4.65 × 10^−4^
						Dominant	1.84 (1.36–2.49)	1.95 (1.40–2.72)	7.99 × 10^−5^
						Recessive	1.45 (1.06–1.98)	1.41 (1.00–2.01)	0.050

Male	Control	118 (20.2)	288 (49.4)	177 (30.4)	0.966		1	1	
	Patient	73 (23.5)	149 (48.1)	88 (28.4)	0.521	Allelic	1.11 (0.91–1.35)	1.18 (0.79–1.75)	0.411
						Additive 1	1.24 (0.84–1.83)	1.18 (0.94–1.47)	0.142
						Additive 2	1.04 (0.76–1.44)	1.39 (0.89–2.17)	0.142
						Dominant	1.09 (0.81–1.49)	1.24 (0.88–1.75)	0.223
						Recessive	1.21 (0.87–1.69)	1.25 (0.86–1.82)	0.237

^#^ *P* value and ORs (95% CI) were adjusted by age.

## References

[1] Tano Y. (2002). Pathologic myopia: where are we now?. *The American Journal of Ophthalmology*.

[2] Vitale S., Ellwein L., Cotch M. F., Ferris F. L., Sperduto R. (2008). Prevalence of refractive error in the United States, 1999–2004. *Archives of Ophthalmology*.

[3] Kempen J. H., Mitchell P., Lee K. E. (2004). The prevalence of refractive errors among adults in the United States, Western Europe, and Australia. *Archives of Ophthalmology*.

[4] Sawada A., Tomidokoro A., Araie M., Iwase A., Yamamoto T. (2008). Refractive errors in an elderly Japanese population: the Tajimi study. *Ophthalmology*.

[5] He M., Zheng Y., Xiang F. (2009). Prevalence of myopia in urban and rural children in mainland china. *Optometry and Vision Science*.

[6] Foster P. J., Hee J., Tielsch J. M., Johnson G. J., Seah S. K. L. (2000). Prevalence and risk factors for refractive errors in adult Chinese in Singapore. *Investigative Ophthalmology and Visual Science*.

[7] Xu L., Li J., Cui T. (2005). Refractive error in urban and rural adult Chinese in Beijing. *Ophthalmology*.

[8] Curtin B. J., Karlin D. B. (1971). Axial length measurements and fundus changes of the myopic eye. *American Journal of Ophthalmology*.

[9] McBrien N. A., Gentle A. (2003). Role of the sclera in the development and pathological complications of myopia. *Progress in Retinal and Eye Research*.

[10] Saw S. M., Gazzard G., Shin-Yen E. C., Chua W. H. (2005). Myopia and associated pathological complications. *Ophthalmic and Physiological Optics*.

[11] He M., Zeng J., Liu Y., Xu J., Pokharel G. P., Ellwein L. B. (2004). Refractive error and visual impairment in urban children in southern China. *Investigative Ophthalmology and Visual Science*.

[12] Dirani M., Chamberlain M., Shekar S. N. (2006). Heritability of refractive error and ocular biometrics: the genes in myopia (GEM) twin study. *Investigative Ophthalmology & Visual Science*.

[13] Lopes M. C., Andrew T., Carbonaro F., Spector T. D., Hammond C. J. (2009). Estimating heritability and shared environmental effects for refractive error in twin and family studies. *Investigative Ophthalmology and Visual Science*.

[14] Verhoeven V. J., Hysi P. G., Wojciechowski R. (2013). Genome-wide meta-analyses of multiancestry cohorts identify multiple new susceptibility loci for refractive error and myopia. *Nature Genetics*.

[15] Cáliz R., Canet L. M., Lupiañez C. B. (2013). Gender-specific effects of genetic variants within Th1 and Th17 cell-mediated immune response genes on the risk of developing rheumatoid arthritis. *PLoS ONE*.

[16] Perez-Becerril C., Morris A. G., Mortimer A., McKenna P. J., de Belleroche J. (2014). Allelic variants in the zinc transporter-3 gene, SLC30A3, a candidate gene identified from gene expression studies, show gender-specific association with schizophrenia. *European Psychiatry*.

[17] Saw S. M., Carkeet A., Chia K. S., Stone R. A., Tan D. T. H. (2002). Component dependent risk factors for ocular parameters in Singapore Chinese children. *Ophthalmology*.

[18] You Q. S., Wu L. J., Duan J. L. (2012). Factors associated with myopia in school children in China: the Beijing Childhood Eye Study. *PLoS ONE*.

[19] Khalil A. M., Guttman M., Huarte M. (2009). Many human large intergenic noncoding RNAs associate with chromatin-modifying complexes and affect gene expression. *Proceedings of the National Academy of Sciences of the United States of America*.

[20] Guttman M., Amit I., Garber M. (2009). Chromatin signature reveals over a thousand highly conserved large non-coding RNAs in mammals. *Nature*.

[21] Talkowski M. E., Ernst C., Heilbut A. (2011). Next-generation sequencing strategies enable routine detection of balanced chromosome rearrangements for clinical diagnostics and genetic research. *American Journal of Human Genetics*.

[22] Huarte M., Guttman M., Feldser D. (2010). A large intergenic noncoding RNA induced by p53 mediates global gene repression in the p53 response. *Cell*.

[23] Loewer S., Cabili M. N., Guttman M. (2010). Large intergenic non-coding RNA-RoR modulates reprogramming of human induced pluripotent stem cells. *Nature Genetics*.

[24] Wang K. C., Yang Y. W., Liu B. (2011). A long noncoding RNA maintains active chromatin to coordinate homeotic gene expression. *Nature*.

[25] Gupta R. A., Shah N., Wang K. C. (2010). Long non-coding RNA HOTAIR reprograms chromatin state to promote cancer metastasis. *Nature*.

[26] Burd C. E., Jeck W. R., Liu Y., Sanoff H. K., Wang Z., Sharpless N. E. (2010). Expression of linear and novel circular forms of an INK4/ARF-associated non-coding RNA correlates with atherosclerosis risk. *PLoS genetics*.

